# Cardiac Dose and Survival Outcomes Following Stereotactic Body Radiation Therapy for Primary and Metastatic Lung Tumors: A Substructure-Based Analysis

**DOI:** 10.1016/j.adro.2026.102059

**Published:** 2026-04-15

**Authors:** Goda Kalinauskaite, Samuel Füchtbauer, Kerstin Rubarth, Thanh-Thao Nguyen, Larissa Silvana Kilian, Felix Mehrhof, Daniel Zips, Carolin Senger

**Affiliations:** a Charité – Universitätsmedizin Berlin, Corporate Member of Freie Universität Berlin and Humboldt Universität zu Berlin, Department of Radiation Oncology, Berlin, Germany; b Berlin Institute of Health at Charité – Universitätsmedizin Berlin, Berlin, Germany; c DKTK, Charité - Universitätsmedizin Berlin, German Cancer Consortium (DKTK), partner site Berlin, and German Cancer Research Center (DKFZ) Heidelberg, Germany; National Center Tumor Diseases (NCT), partner site Berlin, and German Cancer Research Center (DKFZ) Heidelberg, Germany; d Charité – Universitätsmedizin Berlin, Corporate Member of Freie Universität Berlin and Humboldt Universität zu Berlin, Institut of Biometry and Clinical Epidemiology, Berlin, Germany

## Abstract

**Purpose:**

This study investigates the association between radiation dose to the heart base and other cardiac substructures and overall survival (OS) following stereotactic body radiation therapy (SBRT) for central and ultracentral lung tumors.

**Methods and Materials:**

A retrospective analysis was performed on patients treated within the SBRT registry (2011-2023). Autosegmentation was used to contour the whole-heart and cardiac substructures, whereas the heart base—overlapping the ascending aorta and including coronary origins and the sinoatrial node—was manually contoured. Mean (Dmean) and near-maximum doses (D0.03, highest dose to 0.03 cm^3^) were converted to equivalent 2 Gy fractions (EQD₂, α/β = 3 Gy). Optimal dose cut-points were defined to stratify patients into high- and low-dose groups. Cox regression models, adjusted for age, sex, tumor type, and volume, assessed the association between cardiac dose and OS; additional sensitivity analyses included bronchial Dmax.

**Results:**

Among 82 treated patients, 66 were evaluable for dosimetry of cardiac structures and survival analysis. Median age was 67 years; 68% were male. Most lesions were metastases (62.5%) and evenly split between central (54.5%) and ultracentral (45.5%) locations. Higher EQD₂ Dmean to the left atrium (hazard ratio [HR], 2.89; *P* = .04) and superior vena cava (HR, 2.56; *P* = .03), and higher D0.03 to the superior vena cava (HR, 3.12; *P* = .004) and right ventricle (HR, 2.23; *P* = .04) were independently associated with worse OS. After sensitivity analysis, associations for the superior vena cava remained significant, whereas others were attenuated to statistical trends. A higher mean dose to the heart base showed a strong trend toward reduced survival (HR, 3.70; *P* = .05).

**Conclusions:**

Higher radiation doses to the left atrium, superior vena cava, and right ventricle were independently associated with worse OS after SBRT for central and ultracentral lung tumors, underscoring the importance of cardiac-substructure–specific dose constraints in SBRT planning. Moreover, a clear trend toward reduced survival with higher doses to the base of the heart underscores the importance of this region as a potential target for future dose-sparing strategies.

## Introduction

Patients with central and ultracentral lung tumors are at increased risk for severe toxicity and mortality following stereotactic body radiation therapy (SBRT), particularly due to proximity to critical central structures such as the bronchial tree.^1^^,^^2^ The HILUS trial reported grade ≥3 toxicity in 36.9% of patients treated with 8 × 7 Gy, with most severe events occurring in tumors located ≤1 cm from the trachea or main bronchi.^1^ In a subsequent expanded analysis combining prospective and retrospective Nordic cohorts, 13% experienced grade 5 events, with bronchopulmonary bleeding being the predominant cause.^3^ Tumor compression of the tracheobronchial tree and high near-maximum doses to the mainstem or intermediate bronchi were identified as independent risk factors for fatal bleeding. Across other studies, reported rates of grade ≥3 toxicity range from 12% to 34%.^2^^,^^4^, ^5^, ^6^ Complications included severe airway inflammation, bronchial necrosis, and fatal hemoptysis, particularly in cases where tumors encased or compressed central bronchi or vascular structures.

In conventionally fractionated radiation therapy, increasing evidence points to a correlation between cardiac dose and decreased survival. In the RTOG 0617 trial, increased heart V5 (volume receiving ≥5 Gy) was significantly associated with reduced overall survival (OS) in patients with locally advanced non–small cell lung cancer (NSCLC).^7^ Building on this, McWilliam et al^8^ applied a novel voxel-based analysis and identified the base of the heart—an anatomic region that overlaps with the aorta and includes the origin of the coronary arteries and sinoatrial node —as a particularly dose-sensitive area, where even moderate radiation exposure (median 16.3 Gy) significantly impacted 12-months OS. These findings were robustly validated in the external RTOG0617 and PET-plan trial data sets, underscoring the clinical importance of minimizing dose to the heart base during thoracic radiation therapy.^9^

The limiting dose to cardiac substructures in SBRT remains less explored. However, several mostly retrospective studies have investigated individual cardiac substructures in this context. For example, some studies reported a correlation between dose to the superior vena cava and non–cancer-related death,^10^^,^^11^ whereas others found associations between ventricular dose and overall or non–cancer-specific survival.^12^^,^^13^ The base of the heart has not yet been specifically analyzed in this setting.

In SBRT, where treatment volumes are small, using mean or maximum heart dose may miss relevant risks. Artificial intelligence (AI)-based autosegmentation now enables precise contouring of cardiac substructures. Defining and validating constraints for these regions could improve SBRT safety and planning quality. The objective of the present study was to assess the relationship between radiation dose to the heart and its substructures—including the base of the heart as defined by McWilliam et al^8^—and OS in patients with central or ultracentral NSCLC or lung metastases treated with SBRT. We aimed to determine whether higher doses to this critical region contribute to reduced survival, thereby providing a rationale for more stringent cardiac sparing in thoracic SBRT planning.

## Methods and Materials

This retrospective study was approved by the local ethics committee (EA1/233/18). From 2020 onward, all patients were prospectively enrolled in an SBRT registry (EA1/037/20). We identified all patients treated with SBRT using the CyberKnife VSI Radiosurgery System (Accuray Inc) for central or ultracentral primary lung carcinoma or lung metastases between September 2011 and November 2023. Patients were excluded if they had metastases located outside the lung (eg, bone), small cell or neuroendocrine lung carcinoma, peripheral lesions, or missing planning computed tomography (CT) data required for dosimetric analysis.

Tumors were classified as central or ultracentral based on their anatomic relationship to central thoracic structures, following criteria established in previous studies.^14^ Central tumors were defined as those with gross tumor volumes (GTVs) within 2 cm of the proximal bronchial tree or planning target volumes (PTVs) contacting the mediastinal or pericardial pleura. Ultracentral tumors had PTVs directly abutting or overlapping the proximal bronchial tree, trachea, mainstem bronchi, esophagus, or pulmonary vessels.

## Treatment planning

All patients underwent a planning CT scan (slice thickness 1 mm) in the supine position, and contrast medium was administered for tumors abutting the mediastinum. Motion management relied on 3 tracking approaches, Synchrony fiducial tracking after gold marker implantation, Xsight lung tracking, and spine tracking using an internal target volume generated from 4-dimensional CT if motion tracking was not feasible due to lesion characteristics, poor visualization, or patient comorbidities. The GTV was contoured on all axial slices of the planning CT, using lung window settings to include all spiculae and the soft-tissue window for areas abutting the mediastinum. To compensate for setup variability and residual motion, a 5- to 7-mm margin was added, generating the PTV.

Dose prescription at the CyberKnife was inhomogeneous, with the 70% isodose line covering the PTV. Fractionation regimens were selected on an individual basis, considering lesion type (primary tumor vs metastasis), tumor size, anatomic relationship to central thoracic structures, and proximity to organs at risk, reflecting evolving clinical practice over the treatment period from 2011 to 2023. Treatment planning was performed using MultiPlan (Accuray) with the Ray-Trace algorithm until June 2019. From June 2019 onward, planning was carried out in Precision using the Monte Carlo algorithm. Dose constraints for organs at risk were applied according to Benedict et al^15^ until 2021, and from 2021 onward according to Timmerman et al.^16^

### Segmentation of heart substructures

For all patients, the planning CTs were retrospectively imported into the autosegmentation software ART-Plan (TheraPanacea). Which performed an AI-based automatic contouring of the whole heart and following substructures: right and left atria, right and left ventricles, ascending aorta, superior vena cava, pulmonary artery, and the left anterior descending coronary artery.

**Figure 1:**
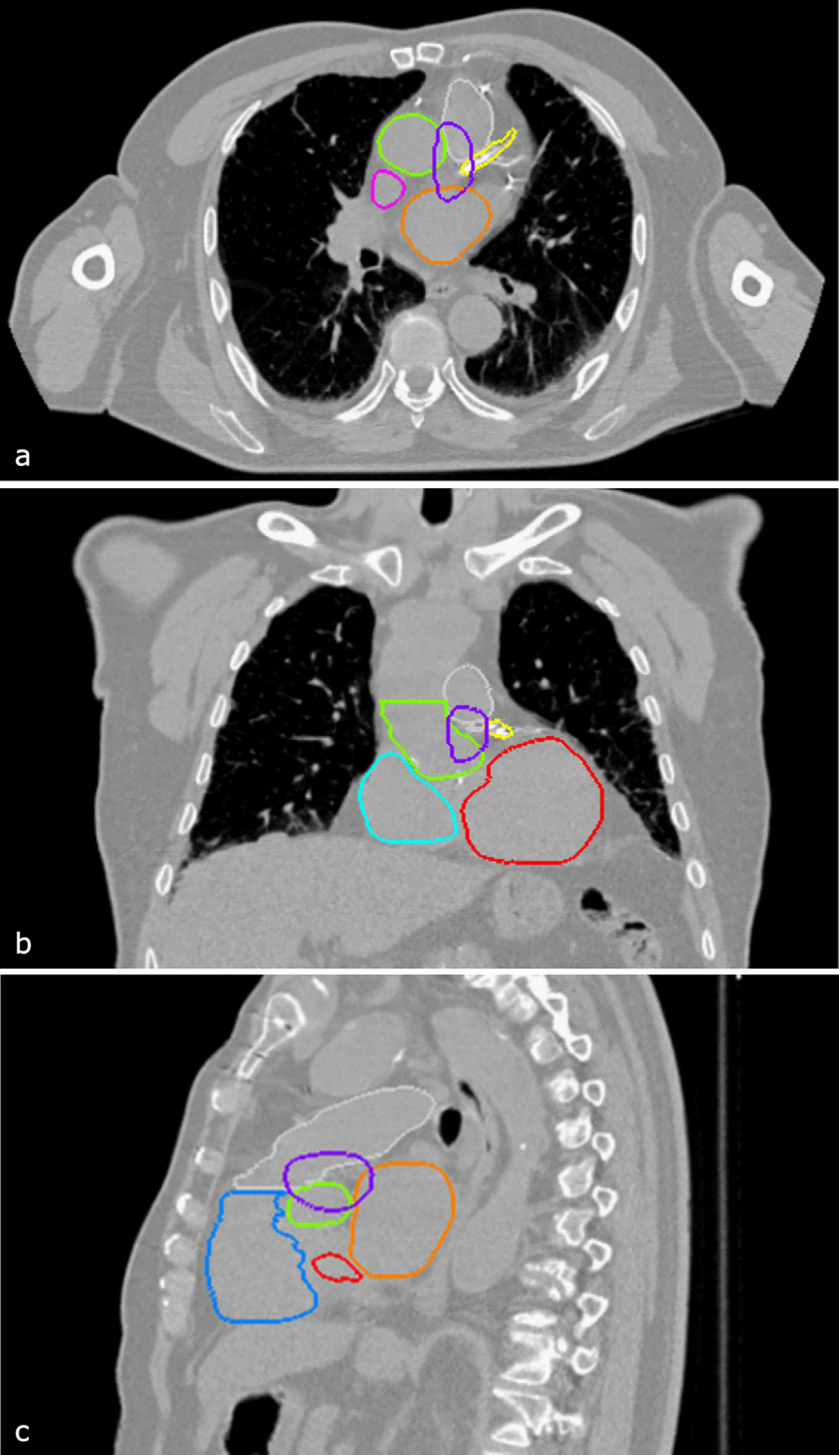
Example of base of the heart contouring (violet) in axial (a), coronal (b), and sagittal (c) planes. Other depicted cardiac substructures include the left atrium (orange), right ventricle (blue), left ventricle (red), ascending aorta (green), and superior vena cava (pink).

The region referred to as the “base of the heart” was contoured according to the anatomic description by McWilliam et al,^8^ who localized this dose-sensitive area near the origin of the coronary arteries and the sinoatrial node. As this region does not correspond to a standard anatomic structure, a semi-automated workflow was developed. The left anterior descending coronary artery, automatically segmented by ART-Plan, was used to locate the origin of the left main coronary artery in axial CT slices. At this level, a 1-cm diameter sphere was manually drawn using the 3-dimensional brush tool. The final base structure was then generated using the "structure margin" function, with asymmetric expansion values in each spatial direction: 0.6 cm (cranial), 2.0 cm (caudal), 1.5 cm (anterior), 1.1 cm (posterior), 1.3 cm (right), and 0.1 cm (left). This method yielded a volume closely resembling the region described in McWilliam et al's^8^ graphical model (Fig. 1).

### Data collection and analysis

We collected demographic data from patient records (age, sex, Karnofsky Performance Status [KPS], cardiac comorbidities), tumor characteristics (location, histology, stage), treatment details (dose, fractionation, tracking modality), and dosimetric parameters such as mean (Dmean) and near-maximum (D0.03, defined as the highest dose to 0.03 cm^3^ of the structure) dose to the PTV, heart, and cardiac substructures, including the base of the heart. Doses to the heart and substructures were recalculated as EQD2 values using an α/β ratio of 3 Gy. Survival status and follow-up duration were documented and cross-checked using data from the Regional Cancer Registry of Brandenburg and Berlin.

Baseline characteristics and dosimetric parameters of the study population were summarized using appropriate descriptive statistics. Survival time and status were defined based on follow-up data. To assess the association between cardiac radiation exposure and OS, radiation dose metrics (EQD2 for Dmean and D0.03) were evaluated for each cardiac substructure. Optimal dose cut-points were identified using the maxstat package in R, which determines the threshold that maximizes survival separation. Based on these cut-points, patients were stratified into low-dose and high-dose groups.

Survival differences between groups were visualized using Kaplan–Meier curves. Cox proportional hazards models adjusted for age, sex, lesion type (primary lung tumor vs metastasis), and tumor burden (volume of treated lesions) were used to estimate hazard ratios (HRs). As an additional sensitivity analysis, the maximum dose to the main bronchus/trachea (Dmax) was additionally included as a covariate to assess potential confounding by bronchial irradiation. Due to missing or inconsistent documentation, KPS and cardiac comorbidities were not included as covariates to avoid bias and preserve statistical power. Given the exploratory nature of the analysis, no correction for multiple comparisons was applied, and *P* values should be interpreted as hypothesis-generating. All analyses were conducted in R.

## Results

A total of 281 patients received thoracic SBRT between January 2011 and December 2023. After applying exclusion criteria, 48 patients were excluded, due to lesion type. Additionally, 122 patients with peripheral lesions and 29 patients with missing planning CT data or treatment plans were excluded. This resulted in 82 patients with central or ultracentral NSCLC (37.5%) or lung metastases (62.5%). Due to technical limitations in cardiac substructure segmentation, 16 of these patients were available only for whole-heart dosimetric analysis. The final analysis cohort for cardiac substructure dose evaluation included 66 patients.

The patient, tumor, and treatment characteristics are presented in Table 1. The majority of tumors were located either centrally (54.5%) or ultracentrally (45.5%). Treatment was most commonly delivered in 3 fractions, applied in 33 patients (37.5%), followed by a single fraction in 28 patients (31.8%), 4 fractions in 12 patients (13.6%), and 5 or more fractions in 15 patients (17.1%). The median GTV volume was 10.1 cm^3^ [IQR: 5.7-21.6]. Median follow-up was 13.9 months (first quartile: 3.8 months, third quartile: 26.4 months).

**Table 1:** Patient, tumor and treatment characteristics

Characteristic	Value
Demographics	
Age, y (median, [IQR])	67 [59, 76]
Sex, no. (%)	
Male	60 (68.2)
Female	28 (31.8)
Karnofsky Performance Status (median [IQR])	80 [70, 90]
Cardiac comorbidities, no. (%)	
Yes	48 (54.5)
No	30 (31.1)
Missing	10 (11.4)
Smoking status	
Current smoker	9 (10.2)
No	17 (19.3)
Unknown	62 (70.5)
Tumor characteristics	
Lesion type, no. (%)	
NSCLC	33 (37.5)
Lung metastases	55 (62.5)
Location, n (%)	
Central	48 (54.5)
Ultracentral	40 (45.5)
GTV volume, cm^3^ (median [IQR])	10.1 [5.7, 21.6]
Primary diagnosis (in case of lung metastasis), no. (%)	
Colorectal cancer	9 (10.2)
Sarcoma	9 (10.2)
Malignant melanoma	7 (8.0)
Head and neck carcinoma	7 (8.0)
Renal cell carcinoma	7 (8.0)
NSCLC	7 (8.0)
Prostate cancer	2 (2.3)
Other	7 (8.0)
Number of lesions treated, no. (%)	
1	75 (85.2)
2-3	11 (12.5)
4-5	2 (2.2)
Treatment characteristics	
Total dose across various fractionation regimens (median [IQR])	
1 fraction	25 [24, 25.25]
3-5 fractions	45 [39, 48]
8 fractions	60 [60, 60]
Fractionation regimen (fractions × dose per fraction, Gy), no. (%)	
1 × 17-26 Gy	28 (31.8)
3 × 8-20 Gy	33 (37.5)
4 × 11.5-12.5 Gy	12 (13.6)
5 × 6-10.5 Gy	11 (12.5)
8 × 7.5 Gy	4 (4.5)
Isodose (median [IQR])	70 [70, 70]
Main bronchus/trachea Dmax (EQD2, α/β = 3; median [IQR])	69.72 [15.74, 102.72]
PTV Dmean, Gy (median [IQR])	49.11 [29.35, 55.68]
PTV Dmean, Gy (BED, α/β = 10; median [IQR])	123.56 [108.75, 148.18]
PTV coverage, % (median [IQR])	98.30 [96.38, 99.27]

*Abbreviations:* BED = biologically effective dose; Dmean = mean dose; EQD2 = equivalent dose in 2-Gy fractions; GTV = gross tumor volume; NSCLC = non–small cell lung cancer; PTV = planning target volume.

**Figure 2:**
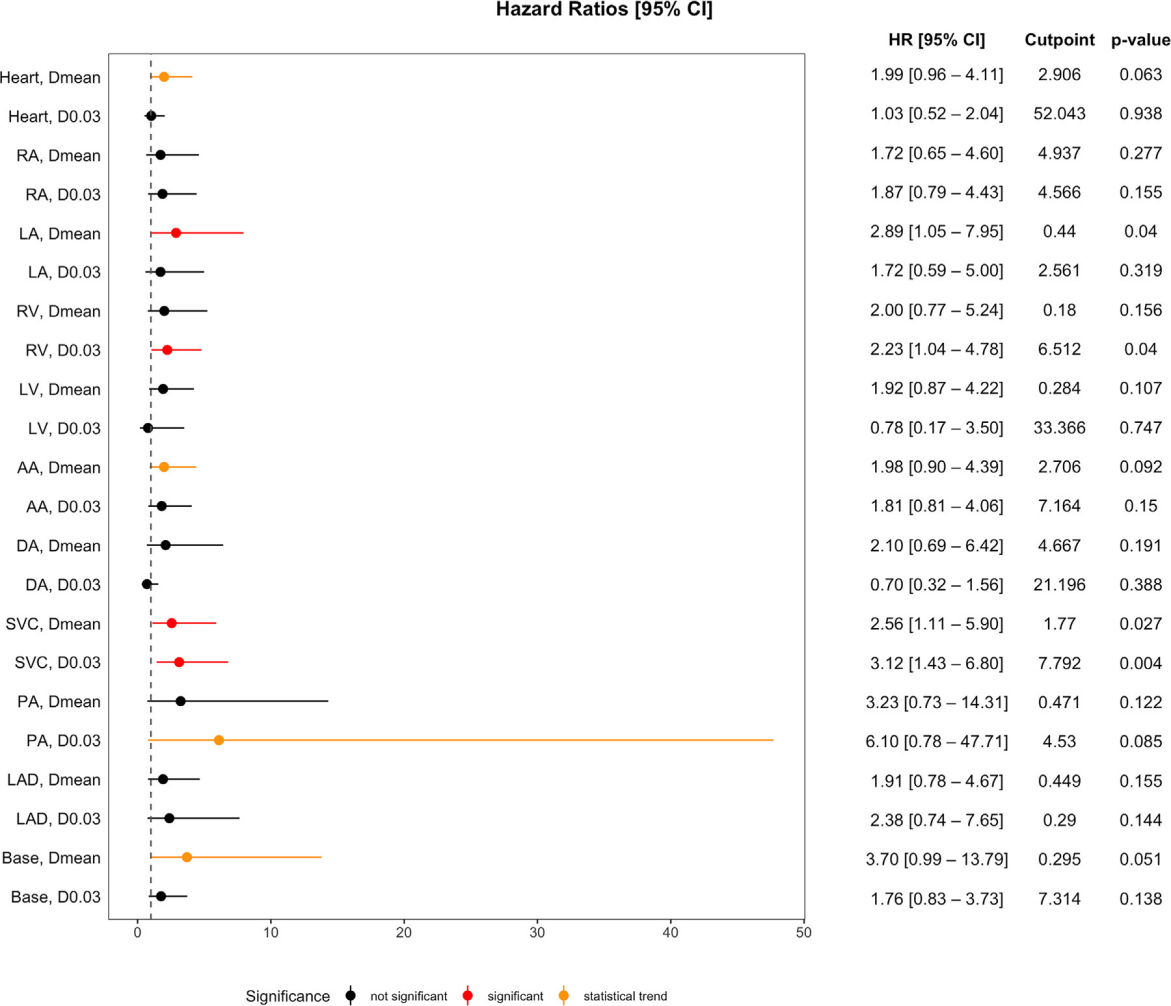
Hazard ratios (HRs) for overall survival (OS) by EQD₂ dose to cardiac substructures. *Abbreviations:* AA = ascending aorta; Base = base of the heart; DA = descending aorta; RA = right atrium; LA = left atrium; LAD = left anterior descending artery; LV = left ventricle; PA = pulmonary artery; RV = right ventricle; SVC = superior vena cava.

Adjusted Cox models showed that several cardiac substructures exhibited a significant association between higher dose exposure—based on identified cut-points—and poorer OS (Fig. 2). Higher EQD₂ Dmean to the left atrium (HR, 2.89; *P* = .04), and superior vena cava (HR, 2.56; *P* = .03), as well as higher D0.03 to the superior vena cava (HR, 3.12; *P* = .004) and right ventricle (HR, 2.23; *P* = .04), each independently predicted significantly poorer OS. Notably, a higher Dmean to the base of the heart showed a strong trend toward worse OS (HR, 3.7; *P* = .05). The cut-point values, as well as the corresponding median dose values for patients in the low- and high-dose groups (below and above the cut-point), are presented in the Table 2.

**Table 2:** Median applied dose values to various cardiac structures

Structure	Overall (median [IQR])	Below cut-point (median [IQR])	Above cut-point (median [IQR])	Cut-point
Heart, Dmean	1.26 [0.55, 3.33]	0.70 [0.37, 1.29]	4.50 [3.84, 5.19]	2.906
Heart, D0.03	48.81 [10.42, 96.15]	10.51 [6.02, 22.93]	96.71 [78.09, 134.44]	52.043
RA, Dmean	1.11 [0.35, 2.47]	0.82 [0.27, 1.67]	6.40 [5.54, 8.09]	4.937
RA, D0.03	6.30 [2.84, 11.74]	1.68 [0.47, 2.90]	9.66 [6.93, 17.85]	4.566
LA, Dmean	1.22 [0.42, 4.57]	0.19 [0.12, 0.28]	2.17 [1.06, 5.63]	0.440
LA, D0.03	8.53 [2.86, 22.66]	0.45 [0.28, 1.51]	12.19 [5.53, 25.94]	2.561
RV, Dmean	0.77 [0.17, 2.21]	0.09 [0.05, 0.13]	1.51 [0.65, 2.86]	0.180
RV, D0.03	5.40 [2.09, 9.92]	2.38 [0.25, 3.92]	11.97 [8.70, 17.95]	6.512
LV, Dmean	0.42 [0.11, 2.44]	0.08 [0.04, 0.15]	1.84 [0.70, 3.19]	0.284
LV, D0.03	5.99 [0.58, 13.96]	4.28 [0.34, 11.45]	64.28 [44.41, 79.99]	33.366
AA, Dmean	2.19 [0.99, 4.65]	1.15 [0.47, 2.00]	5.95 [4.40, 7.73]	2.706
AA, D0.03	8.05 [4.81, 14.37]	4.39 [3.50, 5.50]	13.73 [10.42, 22.34]	7.164
SVC, Dmean	2.51 [0.60, 5.46]	0.55 [0.20, 0.96]	4.85 [2.90, 11.32]	1.770
SVC, D0.03	5.36 [2.01, 10.97]	2.76 [0.77, 5.07]	17.65 [9.93, 49.64]	7.792
PA, Dmean	2.80 [1.14, 5.01]	0.22 [0.13, 0.34]	3.25 [1.88, 5.98]	0.471
PA, D0.03	15.63 [7.95, 60.38]	1.97 [1.28, 2.34]	19.62 [12.78, 81.60]	4.530
LAD, Dmean	1.40 [0.39, 3.63]	0.12 [0.06, 0.26]	2.93 [1.10, 5.72]	0.449
LAD, D0.03	4.08 [1.16, 8.36]	0.13 [0.04, 0.19]	5.53 [1.97, 9.74]	0.290
Base, Dmean	2.29 [0.54, 4.70]	0.17 [0.06, 0.24]	3.21 [1.73, 4.90]	0.295
Base, D0.03	6.76 [1.92, 12.17]	2.51 [0.59, 4.59]	12.61 [10.41, 15.09]	7.314

*Abbreviations:* AA = ascending aorta; Dmean = mean dose; LA = left atrium; LAD = left anterior descending artery; LV = left ventricle; RA = right atrium; RV = right ventricle; SVC = superior vena cava; PA = pulmonary artery.

All dose values are in equivalent dose in 2-Gy fractions, with error bars representing 1 standard deviation.

In univariable analysis, higher maximum dose to the main bronchus/trachea was slightly associated with worse OS (HR, 1.002; 95% CI, 0.999-1.006; *P* = .18), but this small association was not maintained after multivariable adjustment. Bronchial Dmax showed only weak correlations with cardiac dose metrics. After inclusion of bronchial Dmax in multivariable models, dose–outcome associations for the superior vena cava remained significant, whereas associations for the left atrium and right ventricle were attenuated to statistical trends, and the association for the base of the heart mean dose only slightly exceeded our prespecified trend-level significance of 10% (HR, 3.06; 95% CI, 0.88-11.74; *P* = .104; Fig. E1).

**Figure 3:**
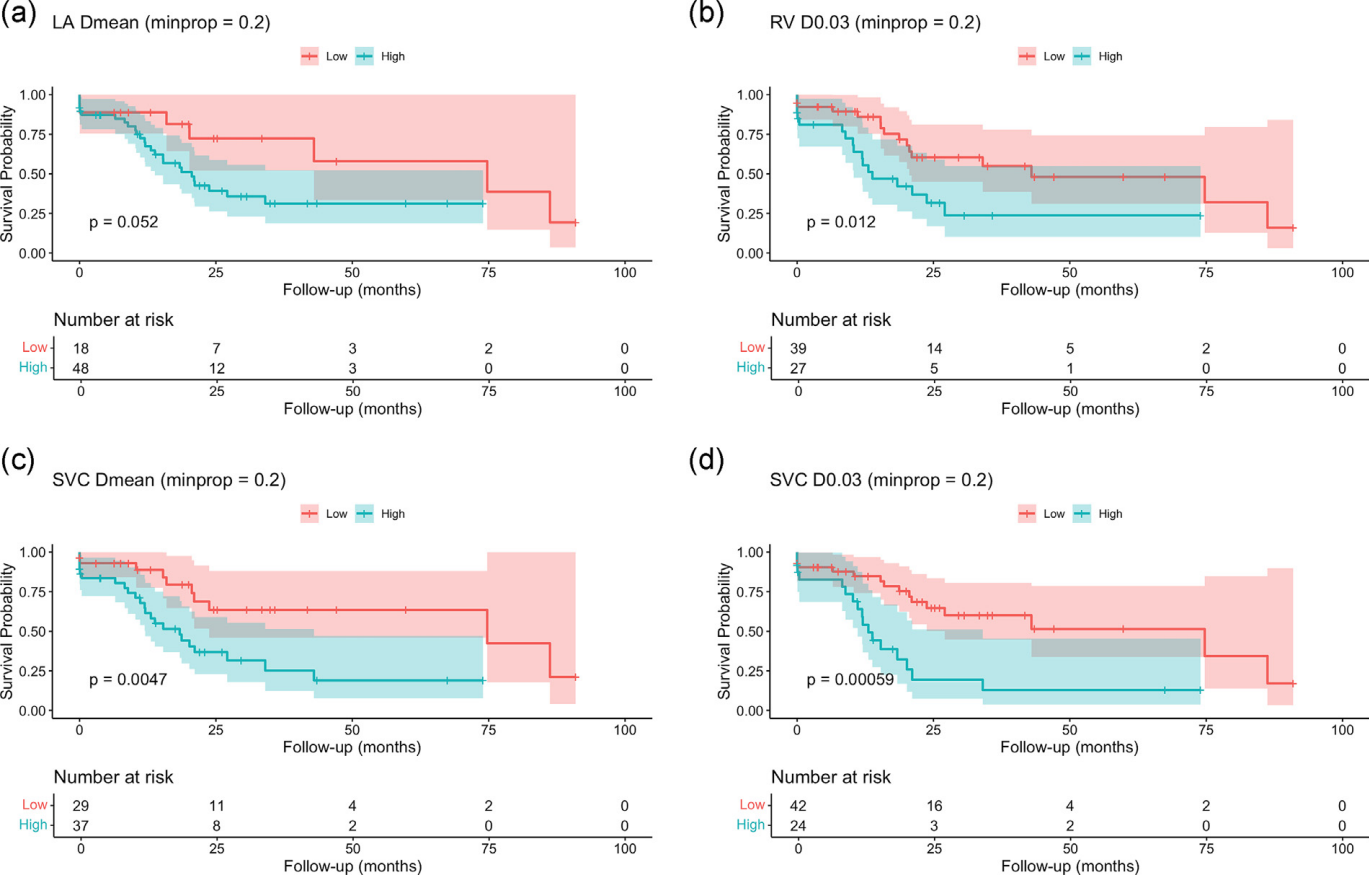
Kaplan–Meier estimates of overall survival (OS) by cardiac substructure dose groups: (A) left atrium (LA) mean dose, (B) right ventricle (RV) D0.03, (C) superior vena cava (SVC) mean dose, (D) superior vena cava D0.03.

The median and 12 months OS for entire population was 20.6 months (IQR, 15.3-43.0 months) and 67.5% (95% CI, 57.3%-78.7%), respectively. Kaplan–Meier curves for OS presented in Fig. 3 illustrate OS stratified by low and high-dose groups for cardiac substructures that showed a significant association between radiation dose and survival. For the left atrium, a higher mean dose (cut-point: 0.44 Gy) was associated with a 1-year OS of 89% (95% CI, 75%-100%) in the low-dose group, compared to 70% (95% CI, 57%-85%) in the high-dose group. For the right ventricle, a higher dose to 0.03 cm^3^ (cut-point: 6.5 Gy) resulted in a 1-year OS of 86% (95% CI, 75%-98%) versus 60% (95% CI, 43%-83%) in the high-dose group. A similar effect was observed for the superior vena cava, where a higher mean dose (cut-point: 1.8 Gy) corresponded to a 1-year OS of 89% (95% CI, 77%-100%) in the low-dose group and 65% (95% CI, 50%-83%) in the high-dose group. When evaluating the maximum point dose to 0.03 cm^3^ of the superior vena cava (cut-point: 7.8 Gy), the 1-year OS was 85% (95% CI, 74%-97%) for the low-dose group and 59% (95% CI, 42%-84%) for the high-dose group.

## Discussion

A growing body of evidence suggests that radiation dose to specific cardiac substructures, rather than to the heart as a whole, may play a critical role in determining noncancer mortality and OS following thoracic radiation therapy. Our data support that certain cardiac substructures, such as the superior vena cava, left atrium and right ventricle, represent critical dosimetric regions influencing OS after SBRT for central and ultracentral lung tumors. In the present study, we observed that higher EQD₂ mean dose to base of the heart showed a trend toward reduced OS (HR, 3.7; *P* = .05). This finding mirrors the voxel-based study by McWilliam et al,^8^ which first demonstrated that the applied dose to the heart base, rather than conventional whole-heart dose constraints, drives mortality in lung cancer radiation therapy. In this study, receiving more than 8.5 Gy to this area had significantly poorer OS (HR, 1.2; *P* < .001), whereas conventional whole-heart parameters such as mean heart dose, V5, and V30 did not show prognostic value.

We also observed, that mean doses to additional upper-cardiac substructures correlated with increased mortality. Increased EQD₂ mean dose to the left atrium (HR, 2.89, *P* = .04), and superior vena cava (HR, 2.56, *P* = .03), each predicted inferior survival. These results are consistent with the retrospective SBRT study by Stam et al^10^ involving 101 stage I–II NSCLC patients, where the maximum EQD₂ dose to the left atrium (median 6.5 Gy; range, 0.009-197; HR, 1.005; *P* = .035) and the dose to 90% of the superior vena cava (median 0.59 Gy; range, 0.003-70; HR, 1.025; *P* = .008) were both independent predictors of non–cancer-related mortality. Their sensitivity analysis localized risk to the upper-cardiac region (atria and great vessels), overlaps with our own findings. Our analysis expands those observations by demonstrating a trend to poorer survival in of heart-base dose for lung SBRT patients. Moreover, to account for potential confounding by bronchial irradiation, we performed a sensitivity analysis adjusting for maximum dose to main bronchus/trachea. Although bronchial dose was not independently associated with OS after adjustment, dose–survival associations for the superior vena cava remained robust, indicating that these findings are not solely driven by bronchial irradiation. Nevertheless, attenuation of some cardiac associations highlights the complex spatial and dosimetric interplay between central thoracic structures and underscores the need for cautious interpretation of causality.

**Figure 4:**
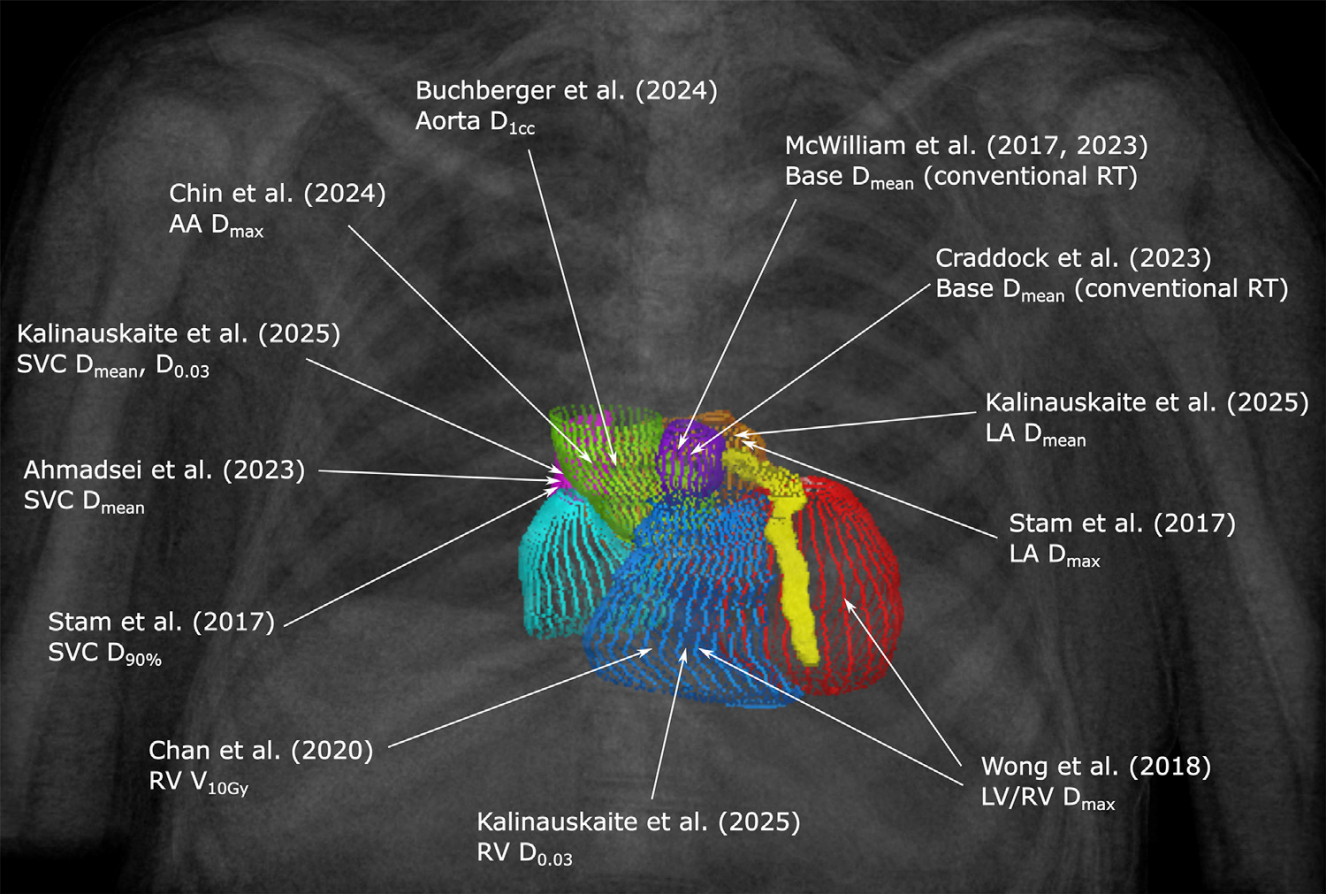
Dose metrics for cardiac substructures associated with decreased overall and non–cancer-specific survival after thoracic stereotactic body radiation therapy (SBRT): literature review and data from present study. To maintain clarity, the pulmonary artery, pulmonary veins, and descending aorta were not included in the figure. *Abbreviations:* AA = ascending aorta (green); Base = base of the heart (violet); LA = left atrium (orange); LV = left ventricle (red); RV = right ventricle (blue); SVC = superior vena cava (pink).

Notably, our results identified a significant association between right ventricular dose metrics and OS. Higher doses to right ventricle were predictive of decreased OS in our cohort, supporting the hypothesis that inferior cardiac structures, including the right ventricle, may also contribute to adverse outcomes. This is consistent with several recent studies: maximum dose to both ventricles has been linked to non–cancer-specific death (univariable analysis *P* = .02; multivariable analysis *P* = .05); a right ventricle V10Gy > 4% was associated with a significantly shorter OS (5.3 vs 2.4 years, *P* = .026); and even low doses (<5 Gy) to inferior heart segments were tied to reduced OS (*P* = .02).^12^^,^^13^^,^^17^ These findings raise important questions about the underlying pathophysiological mechanisms. Two plausible mechanisms are ischemic injury, such as radiation-induced atherosclerosis or perfusion deficits involving the proximal coronary arteries (including the right coronary artery), and radiation-induced arrhythmias due to involvement of the sinus node, atrioventricular node, or components of the conduction system. All of these are anatomically located near the base of the heart. Given the proximity of these critical structures, it remains unclear whether patients are primarily dying from sudden arrhythmic events, progressive heart failure or silent ischemia. Although our study was not designed to disentangle these mechanisms, the spatial pattern of dose–outcome associations suggests that both superior and inferior cardiac substructures may be clinically relevant in thoracic SBRT. The principal results from our cohort and key literature findings are summarized in Fig. 4 and Table E1.^10^, ^11^, ^12^, ^13^^,^^17^, ^18^, ^19^, ^20^, ^21^, ^22^, ^23^

Importantly, our study also observed significant outcome associations despite relatively low absolute doses to some cardiac substructures. This parallels the linear dose–response relationship reported by Darby et al^24^ in breast cancer, where each 1 Gy increase in mean heart dose was associated with a 7.4% rise in major coronary events. Although our endpoint was OS and not cardiac events, the consistent implication is that no clear threshold exists below which cardiac exposure is entirely safe, particularly for anatomically and functionally critical subregions.

Clinical implications: collectively, these findings argue for the integration of cardiac substructure-based planning constraints in thoracic SBRT, even when absolute doses are low. The convergence of evidence across fractionation regimen, patient cohorts, and analytical methodologies underscores the clinical relevance of upper-cardiac substructures, and possibly the base of the heart as priority organs at risk. Future research should aim to prospectively validate these dose–response relationships and to develop robust, consensus-based guidelines for dose constraints to cardiac substructures in thoracic radiation therapy. Routine AI-based autosegmentation of the heart base, left atrium, superior vena cava, and ascending aorta, along with the setting of explicit EQD₂ constraints for each, should become standard in SBRT planning for central and ultracentral lung lesions.

This study has several limitations. First, although AI-based autosegmentation was used for most cardiac substructures, the base of the heart was manually contoured, as no standardized anatomic definition or validated atlas currently exists for this region. This may affect reproducibility and introduces interobserver variability. Second, due to missing or incomplete data, KPS and cardiac comorbidities were not included as covariates in multivariate models, potentially confounding survival outcomes. Third, dose heterogeneity across fractionation regimens and treatment platforms, although accounted for via EQD₂ conversion, may still influence results. Fourth, given the exploratory nature of this analysis and the strong correlations among cardiac dose–volume histogram metrics, no formal correction for multiple comparisons was applied, and the observed associations should be interpreted as hypothesis-generating and requiring independent validation. Fifth, the broader definition of central disease used in this study, extending beyond the original bronchial “no-fly zone,” partly explains the observed heterogeneity in fractionation regimens and supports the relevance of this cohort for cardiac dose analyses. Finally, cause-specific mortality data were unavailable, limiting the ability to distinguish cancer-related from cardiac or treatment-related deaths. However, our findings align with prior studies in conventionally fractionated lung radiation therapy, highlighting upper-cardiac substructures, including the base of the heart, as critical regions. This consistency supports the relevance of these areas for dose-sparing in thoracic SBRT.

## Conclusions

In patients receiving thoracic SBRT, dose–response relationships are driven by cardiac substructures rather than by whole-heart exposure. Elevated EQD₂ doses to upper-cardiac structures, such as the left atrium and superior vena cava, were independently associated with reduced OS. Additionally, there was a trend toward decreased survival with higher doses to the cardiac base, indicating a consistent vulnerability of this region. Conventional whole-heart metrics may therefore underestimate risk in the SBRT setting. Treatment planning should incorporate substructure-specific constraints and employ advanced motion management techniques to spare these high-risk regions without compromising target coverage.

## Disclosures

DZ disclose financial technical and educational support for scientific projects received by the department of radiation oncology Tübingen and Charité (Elekta, Philips, Varian/Siemens, Sennewald, Therapanacea, and PTW). Presentation fees for the mentioned industrial partners were paid to university of Tübingen and Charité. The other authors declare that they have no known competing financial interests or personal relationships that could have appeared to influence the work reported in this paper.

## Appendix Supplementary materials

Figure_E1 Image, application 1

Table_E1 Image, application 2

## Footnotes

Supplementary material associated with this article can be found in the online version at doi:10.1016/j.adro.2026.102059.
