# Supplementary material for: Cardiac Dose and Survival Outcomes Following Stereotactic Body Radiation Therapy for Primary and Metastatic Lung Tumors: A Substructure-Based Analysis
Source: Adv Radiat Oncol. 2026 Apr 15;11(7):102059. doi: 10.1016/j.adro.2026.102059 (PMC13202019; doi:10.1016/j.adro.2026.102059)
Supplement: Figure_E1 [file mmc1.docx]

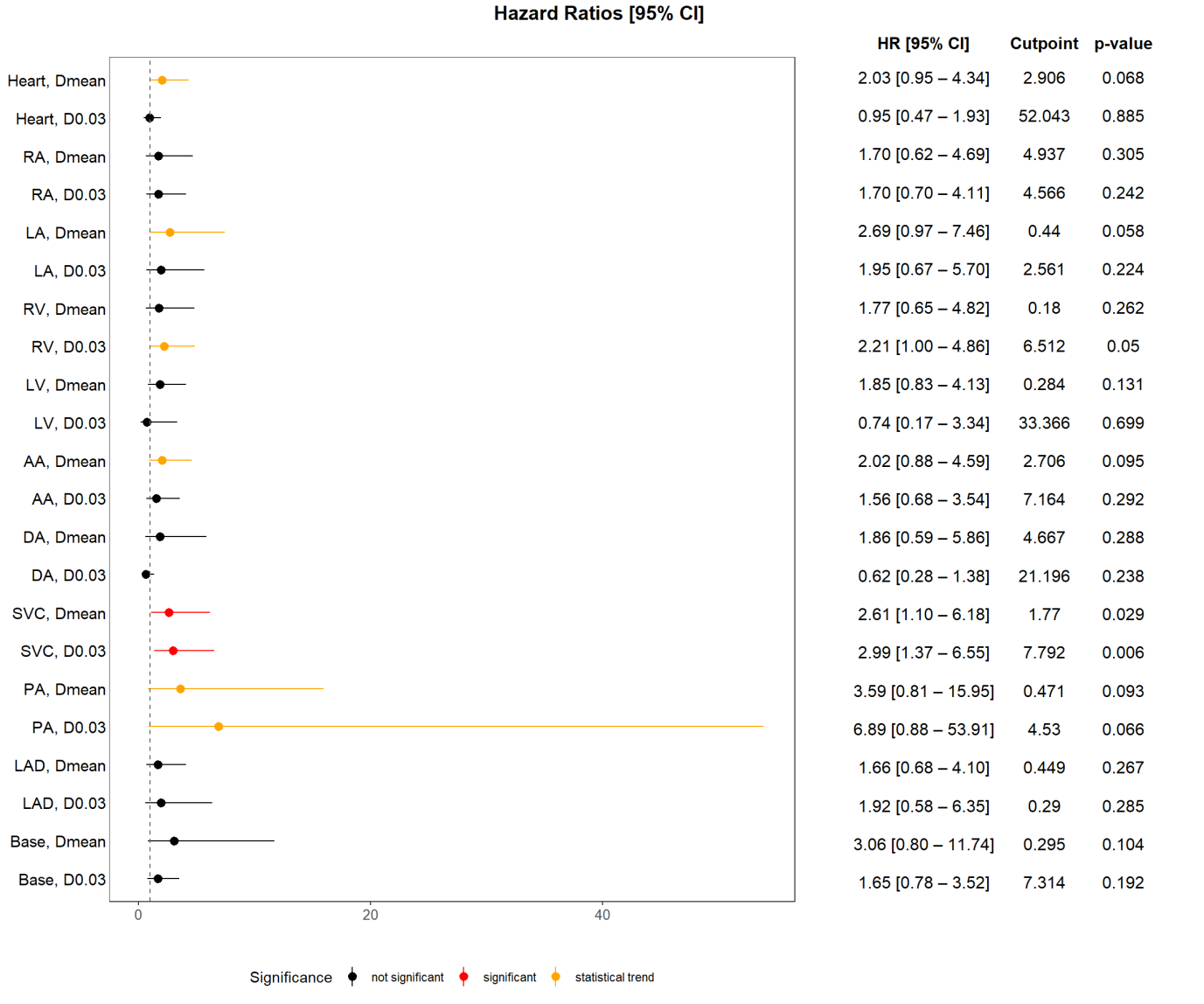


**Figure E1**. Hazard ratios (HR) for overall survival (OS) by EQD₂ dose to cardiac substructures with additional adjustment for bronchial irradiation (main bronchus/trachea Dmax). Abbreviations: Basse = base of the heart, RA = right atrium, LA = left atrium, RV = right ventricle, LV = left ventricle, AA = ascending aorta, DA = descending aorta, SVC = superior vena cava, PA = pulmonary artery, LAD = left anterior descending artery.
