# Supplementary material for: Cardiac Dose and Survival Outcomes Following Stereotactic Body Radiation Therapy for Primary and Metastatic Lung Tumors: A Substructure-Based Analysis
Source: Adv Radiat Oncol. 2026 Apr 15;11(7):102059. doi: 10.1016/j.adro.2026.102059 (PMC13202019; doi:10.1016/j.adro.2026.102059)
Supplement: Table_E1 [file mmc2.docx]

| Table E1. Cardiotoxicity after Stereotactic Body Radiotherapy: Overview of Key Publications | | | | | | | | |
| --- | --- | --- | --- | --- | --- | --- | --- | --- |
| Author (Year) | **Study Type / Sample Size** | **Population** | **Radiotherapy Technique (Dose, Fractionation)** | **Endpoint(s)** | **Follow-up** | **Structures** | **Statistical Method(s)** | **Main Findings** |
| Stam (2017) | Retrospective, multicenter, n = 803 | Early-stage NSCLC | SBRT, 37.5–60 Gy in 3–8 fractions | Non-cancer-specific death | Median 2.9 years | Heart and substructures | Cox proportional hazards models; sensitivity analysis | Left atrium Dmax and SVC D90% associated with non-cancer-specific death (p = 0.035; p = 0.008). Upper heart region dose also linked in sensitivity analysis. |
| Wong (2017) | Retrospective, single-center, n = 189 | Early-stage NSCLC | SBRT, 48–60 Gy in 3–8 fractions | Non-cancer-specific death | Median 2.94 years | Heart and substructures | Cox proportional hazards models | Maximum dose to both ventricles associated with non-cancer-specific death (UVA p = 0.02; MVA p = 0.05). |
| Tembhekar (2017) | Retrospective, single-center, n = 102 | Early-stage NSCLC | SBRT, 40–60 Gy in 3–8 fractions | OS | Median 2.27 years | Heart | Kaplan–Meier; Cox proportional hazards models | No correlation between OS and heart dose parameters. |
| Reshko (2018) | Retrospective, single-center, n = 74 | Early-stage NSCLC/SCLC | SBRT, 40–60 Gy in 4–8 fractions | OS; cardiac events | Mean 2.92 years | Heart and substructures | Mann–Whitney U; Pearson correlation; chi-squared; Kaplan–Meier | No correlation between OS and heart dose parameters. |
| Chan (2019) | Retrospective, single-center, n = 112 | Early-stage NSCLC | SBRT, 50–54 Gy in 3–5 fractions | OS; cardiac events | Median 5.1 years | Heart and substructures | Kaplan–Meier; artificial neural network | Right ventricle V10 Gy > 4% linked to reduced OS (5.3 vs 2.4 years, p = 0.026). |
| Anderson (2021) | Retrospective, single-center, n = 197 | Locally advanced NSCLC | SBRT, 50 Gy/5 fractions; 48 Gy/4 fractions | OS | Median 2.33 years | Heart (4 quadrants & 64 segments) | Cox proportional hazards; AIC | Low doses (< 5 Gy) to inferior heart segments tied to reduced OS (p = 0.02). |
| Liu (2022) | Retrospective, database (SEER), n = 3,256 | Stage I–IIA NSCLC, ≥ 65 y | SBRT vs 3DCRT/IMRT | Cardiovascular events | Median 2 years | Left vs right-sided tumors | Kaplan–Meier; Cox proportional hazards | No difference with SBRT. Left-sided tumors under 3DCRT/IMRT had higher heart failure risk (HR 1.23). |
| Iovoli (2023) | Retrospective, single-center, n = 93 | Central/ultracentral NSCLC | SBRT, 50–60 Gy in 5 fractions | OS; NCS | Median 2.7 years | SAN & AV nodes | Cox proportional hazards | SAN Dmean & Dmax above cutoff associated with OS (p = 0.011; 0.026) and NCS (p = 0.037). |
| Ahmadsei (2023) | Retrospective, single-center, n = 60 | Ultra-central lung tumors | SBRT, 30–60 Gy in 5–12 fractions | OS; PFS; local control; cardiotoxicity; NCS death | Median 2.2 years | Heart and substructures | Kaplan–Meier; Cox proportional hazards | Mean heart dose, SVC, and pulmonary artery doses tied to NCS death (HR 1.2; CI 1.1–1.4; p = 0.0026). |
| Chin (2024) | Prospective, multicenter, n = 117 | Early-stage NSCLC | SBRT, 45–54 Gy in 3–5 fractions | OS; pericardial effusion | Median 2.93 years | Heart and substructures | Kaplan–Meier; Cox proportional hazards | Above-median MHD associated with worse OS (p = 0.00004). Dmax to ascending aorta linked to worse OS in MVA. |
| Buchberger (2024) | Prospective, single-center, n = 89 | Lung tumors ≤ 1 cm from heart/vessels | SBRT, 34–60 Gy in 1–8 fractions | NCS death; OS; toxicity | Median 1.83 years | Heart and substructures | Competing risk regression; Cox proportional hazards | Aorta D1cc significantly linked to NCS death (HR 1.04, p < 0.05). |

**Abbreviations:** NSCLC = non–small cell lung cancer; SCLC = small cell lung cancer; SBRT = stereotactic body radiotherapy; 3DCRT = three‑dimensional conformal radiotherapy; IMRT = intensity‑modulated radiotherapy; OS = overall survival; PFS = progression‑free survival; NCS = non‑cancer‑specific survival; HR = hazard ratio; MHD = mean heart dose; SVC = superior vena cava; SAN = sinoatrial node; AV node = atrioventricular node; Dmax = maximum dose; Dmean = mean dose; D1cc = dose to 1 cm³; Vx = volume receiving ≥ x Gy (e.g., V10 Gy); AIC = Akaike information criterion; CI = confidence interval.
